# Supplementary material for: A Likelihood Approach for Real-Time Calibration of Stochastic Compartmental Epidemic Models
Source: PLoS Comput Biol. 2017 Jan 17;13(1):e1005257. doi: 10.1371/journal.pcbi.1005257 (PMC5240920; doi:10.1371/journal.pcbi.1005257)
Supplement: S1 File — (TAR.GZ) [file pcbi.1005257.s014.tar.gz › HSPH_Online-SI-Revision/Additions/MSS11/n100-extreme-SEITR/n100-extreme-SEITR_NEW2_table4.pdf]

1	newly 3 weeks spec	1057.	1423.13	{1377., 1460.}	34.6386	0.129039	newly 3 weeks cumul	5452.	6049.39	{5993., 6110.}	10.9573	0.0451424	newly 5 weeks spec	382.	750.24	{720., 779.}	96.3979	0.292881	newly 5 weeks cumul	6457.	7829.96	{7773., 7888.}	21.2631	0.0837218	newly 47 weeks cumul	7014.	9788.8	{9724., 9851.}	39.5609	0.144758
2	newly 3 weeks spec	909.	231.61	{212., 254.}	74.5204	0.594905	newly 3 weeks cumul	5060.	1517.25	{1475., 1553.}	70.0148	0.523171	newly 5 weeks spec	300.	63.34	{51., 74.}	78.8867	0.679537	newly 5 weeks cumul	5879.	1700.9	{1665., 1736.}	71.0682	0.538691	newly 54 weeks cumul	6291.	1766.42	{1721., 1808.}	71.9215	0.551701
3	newly 3 weeks spec	1244.	511.44	{478., 540.}	58.8875	0.386462	newly 3 weeks cumul	6664.	2925.39	{2884., 2960.}	56.1016	0.357574	newly 5 weeks spec	463.	167.76	{151., 185.}	63.7667	0.442234	newly 5 weeks cumul	7872.	3385.34	{3334., 3424.}	56.9952	0.366507	newly 56 weeks cumul	8546.	3611.35	{3558., 3659.}	57.7422	0.374121
4	newly 3 weeks spec	1162.	717.61	{679., 752.}	38.2435	0.209606	newly 3 weeks cumul	6474.	3513.1	{3466., 3557.}	45.7352	0.265505	newly 5 weeks spec	412.	296.6	{273., 320.}	28.0097	0.14353	newly 5 weeks cumul	7613.	4271.7	{4224., 4319.}	43.8894	0.250972	newly 54 weeks cumul	8186.	4809.26	{4753., 4865.}	41.2502	0.231012
5	newly 3 weeks spec	334.	0.75	{0., 2.}	99.7754	3.00218	newly 3 weeks cumul	2304.	11.33	{3., 21.}	99.5082	2.40349	newly 5 weeks spec	78.	0.09	{0., 0.}	99.8846	2.80209	newly 5 weeks cumul	2553.	11.66	{3., 21.}	99.5433	2.43668	newly 56 weeks cumul	2676.	11.67	{3., 22.}	99.5639	2.45691
6	newly 3 weeks spec	210.	59.91	{49., 70.}	71.4714	0.548563	newly 3 weeks cumul	1688.	405.66	{380., 433.}	75.968	0.619843	newly 5 weeks spec	40.	15.79	{10., 22.}	60.525	0.421527	newly 5 weeks cumul	1825.	452.4	{426., 483.}	75.211	0.606332	newly 55 weeks cumul	1849.	469.19	{439., 503.}	74.6247	0.596207
7	newly 3 weeks spec	1990.	1596.4	{1552., 1635.}	19.7789	0.0958134	newly 3 weeks cumul	8888.	6818.36	{6759., 6876.}	23.2858	0.115136	newly 5 weeks spec	982.	840.34	{805., 878.}	14.4257	0.0679323	newly 5 weeks cumul	11 272.	8819.52	{8758., 8874.}	21.7573	0.106562	newly 47 weeks cumul	13 516.	10 981.2	{10 913., 11 059.}	18.7538	0.090202
8	newly 3 weeks spec	1262.	472.29	{443., 499.}	62.5761	0.427298	newly 3 weeks cumul	6258.	2711.38	{2662., 2754.}	56.6734	0.363282	newly 5 weeks spec	510.	153.89	{138., 171.}	69.8255	0.521761	newly 5 weeks cumul	7632.	3133.47	{3077., 3184.}	58.943	0.386649	newly 54 weeks cumul	8594.	3339.22	{3277., 3396.}	61.1448	0.41059
9	newly 3 weeks spec	351.	0.56	{0., 2.}	99.8405	3.12867	newly 3 weeks cumul	2373.	7.41	{2., 15.}	99.6877	2.61198	newly 5 weeks spec	96.	0.04	{0., 0.}	99.9583	2.94227	newly 5 weeks cumul	2626.	7.6	{2., 16.}	99.7106	2.64356	newly 56 weeks cumul	2718.	7.61	{2., 16.}	99.72	2.65828
10	newly 3 weeks spec	2417.	1240.82	{1191., 1284.}	48.6628	0.289743	newly 3 weeks cumul	10 500.	5717.87	{5646., 5792.}	45.5441	0.263976	newly 5 weeks spec	1167.	566.87	{532., 595.}	51.425	0.313957	newly 5 weeks cumul	13 414.	7115.6	{7038., 7203.}	46.9539	0.275364	newly 52 weeks cumul	16 196.	8271.62	{8179., 8365.}	48.928	0.291835
11	newly 3 weeks spec	2013.	795.14	{752., 828.}	60.4998	0.403715	newly 3 weeks cumul	9231.	4562.92	{4485., 4623.}	50.5696	0.306033	newly 5 weeks spec	1003.	262.62	{238., 282.}	73.8166	0.582883	newly 5 weeks cumul	11 702.	5283.7	{5209., 5348.}	54.8479	0.345344	newly 51 weeks cumul	13 920.	5633.14	{5556., 5714.}	59.532	0.392915
12	newly 3 weeks spec	1233.	565.04	{533., 591.}	54.1736	0.339184	newly 3 weeks cumul	6317.	2967.18	{2920., 3004.}	53.0287	0.328195	newly 5 weeks spec	542.	212.83	{192., 236.}	60.7325	0.407147	newly 5 weeks cumul	7681.	3526.41	{3482., 3570.}	54.0892	0.338106	newly 52 weeks cumul	8581.	3858.14	{3801., 3906.}	55.0386	0.347185
13	newly 3 weeks spec	1573.	599.66	{558., 636.}	61.8779	0.41932	newly 3 weeks cumul	7548.	3297.75	{3234., 3345.}	56.3096	0.359648	newly 5 weeks spec	706.	206.73	{189., 225.}	70.7181	0.534408	newly 5 weeks cumul	9330.	3857.26	{3798., 3911.}	58.6574	0.383633	newly 53 weeks cumul	10 733.	4158.23	{4092., 4222.}	61.2575	0.411846
14	newly 3 weeks spec	2628.	2177.85	{2121., 2227.}	17.129	0.081678	newly 3 weeks cumul	11 099.	8833.76	{8747., 8911.}	20.4094	0.0991506	newly 5 weeks spec	1313.	1225.16	{1181., 1274.}	6.69002	0.0302327	newly 5 weeks cumul	14 282.	11 694.3	{11 619., 11 760.}	18.1185	0.0868196	newly 46 weeks cumul	17 443.	15 325.2	{15 249., 15 396.}	12.1413	0.0562186
15	newly 3 weeks spec	1421.	203.26	{183., 225.}	85.696	0.845782	newly 3 weeks cumul	6948.	1192.61	{1138., 1236.}	82.8352	0.765612	newly 5 weeks spec	621.	65.08	{54., 77.}	89.5201	0.983714	newly 5 weeks cumul	8459.	1371.73	{1311., 1424.}	83.7838	0.790293	newly 47 weeks cumul	9505.	1455.44	{1392., 1512.}	84.6876	0.815217
16	newly 3 weeks spec	1685.	1376.93	{1332., 1422.}	18.2831	0.0878203	newly 3 weeks cumul	8356.	6357.72	{6284., 6429.}	23.9143	0.118712	newly 5 weeks spec	644.	636.16	{598., 673.}	3.57143	0.0156866	newly 5 weeks cumul	10 017.	7931.2	{7856., 8013.}	20.8226	0.101412	newly 50 weeks cumul	11 020.	9244.74	{9132., 9334.}	16.1094	0.076302
17	newly 3 weeks spec	244.	372.84	{346., 398.}	52.8033	0.183386	newly 3 weeks cumul	2213.	2369.07	{2318., 2414.}	7.05242	0.029545	newly 5 weeks spec	41.	103.	{87., 116.}	151.22	0.39694	newly 5 weeks cumul	2358.	2670.12	{2610., 2728.}	13.2366	0.0539299	newly 56 weeks cumul	2381.	2787.07	{2734., 2850.}	17.0546	0.0683272
18	newly 3 weeks spec	946.	82.48	{71., 96.}	91.2812	1.06295	newly 3 weeks cumul	4981.	561.17	{534., 588.}	88.7338	0.948544	newly 5 weeks spec	314.	21.68	{16., 30.}	93.0955	1.17391	newly 5 weeks cumul	5835.	626.79	{599., 658.}	89.2581	0.969251	newly 55 weeks cumul	6316.	650.71	{616., 688.}	89.6974	0.987418
19	newly 3 weeks spec	2246.	1077.11	{1035., 1120.}	52.0432	0.319351	newly 3 weeks cumul	9933.	5378.53	{5312., 5442.}	45.8519	0.266436	newly 5 weeks spec	1136.	432.19	{396., 467.}	61.9551	0.420475	newly 5 weeks cumul	12 584.	6499.08	{6434., 6565.}	48.3544	0.286982	newly 50 weeks cumul	14 784.	7254.71	{7175., 7328.}	50.9286	0.309189
20	newly 3 weeks spec	688.	2.37	{0., 5.}	99.6555	2.73291	newly 3 weeks cumul	3837.	16.65	{4., 32.}	99.5661	2.47585	newly 5 weeks spec	219.	0.67	{0., 2.}	99.6941	2.90888	newly 5 weeks cumul	4426.	18.57	{4., 36.}	99.5804	2.48955	newly 55 weeks cumul	4693.	19.05	{4., 37.}	99.5941	2.50289
21	newly 3 weeks spec	566.	2.02	{0., 5.}	99.6431	2.77284	newly 3 weeks cumul	3401.	16.3	{4., 32.}	99.5207	2.43698	newly 5 weeks spec	156.	0.39	{0., 1.}	99.75	2.86154	newly 5 weeks cumul	3828.	17.5	{5., 33.}	99.5428	2.45276	newly 56 weeks cumul	3988.	17.76	{5., 34.}	99.5547	2.46535
22	newly 3 weeks spec	1150.	792.88	{756., 833.}	31.0539	0.161774	newly 3 weeks cumul	5834.	3867.36	{3816., 3915.}	33.71	0.17857	newly 5 weeks spec	400.	336.81	{313., 356.}	15.7975	0.0751982	newly 5 weeks cumul	6931.	4719.32	{4674., 4765.}	31.91	0.166931	newly 52 weeks cumul	7573.	5344.58	{5294., 5396.}	29.4259	0.151368
23	newly 3 weeks spec	361.	688.97	{652., 727.}	90.8504	0.280331	newly 3 weeks cumul	2348.	3562.56	{3511., 3613.}	51.7274	0.181038	newly 5 weeks spec	79.	266.1	{242., 287.}	236.835	0.526502	newly 5 weeks cumul	2588.	4256.42	{4197., 4314.}	64.4675	0.216059	newly 53 weeks cumul	2670.	4681.57	{4611., 4747.}	75.3397	0.243849
24	newly 3 weeks spec	617.	19.68	{13., 27.}	96.8104	1.51661	newly 3 weeks cumul	3939.	158.4	{127., 189.}	95.9787	1.40152	newly 5 weeks spec	171.	3.72	{1., 6.}	97.8246	1.79869	newly 5 weeks cumul	4404.	170.61	{135., 201.}	96.126	1.41786	newly 55 weeks cumul	4602.	173.49	{137., 205.}	96.2301	1.42977
25	newly 3 weeks spec	1330.	808.39	{772., 841.}	39.2188	0.21649	newly 3 weeks cumul	7007.	4040.73	{3979., 4096.}	42.333	0.239097	newly 5 weeks spec	443.	328.8	{305., 353.}	25.7788	0.130216	newly 5 weeks cumul	8227.	4886.74	{4815., 4948.}	40.6012	0.226246	newly 51 weeks cumul	8870.	5465.99	{5382., 5548.}	38.3767	0.210284
26	newly 3 weeks spec	920.	1656.67	{1612., 1704.}	80.0728	0.255338	newly 3 weeks cumul	5136.	7372.57	{7295., 7439.}	43.5469	0.156982	newly 5 weeks spec	345.	801.67	{760., 844.}	132.368	0.36588	newly 5 weeks cumul	6057.	9336.78	{9269., 9403.}	54.1486	0.187933	newly 49 weeks cumul	6590.	11 157.4	{11 070., 11 234.}	69.3076	0.228669
27	newly 3 weeks spec	2118.	1533.23	{1479., 1578.}	27.6095	0.14045	newly 3 weeks cumul	9250.	6670.25	{6607., 6739.}	27.8892	0.142013	newly 5 weeks spec	1079.	786.4	{751., 823.}	27.1177	0.137665	newly 5 weeks cumul	11 803.	8556.17	{8493., 8618.}	27.5085	0.139721	newly 49 weeks cumul	14 480.	10 468.1	{10 400., 10 536.}	27.7065	0.140906
28	newly 3 weeks spec	196.	390.91	{367., 426.}	99.4439	0.299141	newly 3 weeks cumul	1632.	2429.69	{2370., 2480.}	48.8781	0.172767	newly 5 weeks spec	32.	113.05	{98., 129.}	253.281	0.545477	newly 5 weeks cumul	1750.	2749.21	{2684., 2811.}	57.0977	0.196094	newly 56 weeks cumul	1781.	2878.22	{2806., 2940.}	61.607	0.208386
29	newly 3 weeks spec	1912.	1039.98	{998., 1077.}	45.6077	0.264647	newly 3 weeks cumul	9043.	5309.44	{5246., 5370.}	41.2867	0.231281	newly 5 weeks spec	849.	402.91	{380., 429.}	52.543	0.324177	newly 5 weeks cumul	11 162.	6361.62	{6307., 6425.}	43.0065	0.244187	newly 53 weeks cumul	12 786.	7034.45	{6961., 7109.}	44.9832	0.259519
30	newly 3 weeks spec	1221.	495.32	{462., 528.}	59.4333	0.392529	newly 3 weeks cumul	6812.	3241.81	{3215., 3316.}	52.4103	0.323025	newly 5 weeks spec	428.	133.78	{119., 148.}	68.743	0.507035	newly 5 weeks cumul	7969.	3634.47	{3611., 3704.}	54.3924	0.341475	newly 56 weeks cumul	8592.	3780.92	{3760., 3859.}	55.9949	0.356952
31	newly 3 weeks spec	1586.	1486.45	{1439., 1534.}	6.2768	0.0282841	newly 3 weeks cumul	7252.	6696.15	{6606., 6768.}	7.66478	0.0346519	newly 5 weeks spec	689.	713.43	{685., 738.}	3.99855	0.0169021	newly 5 weeks cumul	9024.	8440.88	{8353., 8522.}	6.46188	0.0290242	newly 46 weeks cumul	10 290.	9990.26	{9887., 10 105.}	2.91293	0.012855
32	newly 3 weeks spec	2184.	1456.81	{1418., 1503.}	33.2962	0.175957	newly 3 weeks cumul	9583.	6510.25	{6428., 6576.}	32.0646	0.167927	newly 5 weeks spec	1094.	703.9	{673., 735.}	35.6581	0.191824	newly 5 weeks cumul	12 124.	8231.79	{8147., 8308.}	32.1033	0.168172	newly 52 weeks cumul	14 626.	9806.34	{9720., 9895.}	32.9527	0.173645
33	newly 3 weeks spec	643.	53.72	{44., 63.}	91.6454	1.08341	newly 3 weeks cumul	3804.	372.28	{349., 404.}	90.2135	1.01003	newly 5 weeks spec	197.	14.19	{9., 19.}	92.797	1.15938	newly 5 weeks cumul	4382.	413.24	{383., 442.}	90.5696	1.02608	newly 56 weeks cumul	4693.	427.91	{395., 460.}	90.882	1.04076
34	newly 3 weeks spec	883.	585.23	{550., 617.}	33.7225	0.179045	newly 3 weeks cumul	4731.	2772.67	{2722., 2815.}	41.3936	0.232095	newly 5 weeks spec	278.	256.36	{232., 281.}	8.33813	0.038502	newly 5 weeks cumul	5501.	3411.82	{3359., 3463.}	37.9782	0.20749	newly 52 weeks cumul	5915.	3906.69	{3841., 3962.}	33.9528	0.180177
35	newly 3 weeks spec	239.	69.76	{59., 80.}	70.8117	0.538068	newly 3 weeks cumul	1921.	578.17	{550., 608.}	69.9027	0.521806	newly 5 weeks spec	50.	13.33	{9., 18.}	73.34	0.592028	newly 5 weeks cumul	2064.	621.49	{593., 651.}	69.8891	0.521585	newly 56 weeks cumul	2096.	632.34	{604., 665.}	69.8311	0.520755
36	newly 3 weeks spec	2432.	1149.35	{1106., 1196.}	52.7405	0.325697	newly 3 weeks cumul	10 891.	5900.07	{5830., 5966.}	45.8262	0.266226	newly 5 weeks spec	1211.	443.83	{415., 470.}	63.3501	0.436446	newly 5 weeks cumul	13 782.	7060.5	{6994., 7151.}	48.7701	0.290495	newly 52 weeks cumul	16 479.	7786.31	{7685., 7887.}	52.7501	0.325619
37	newly 3 weeks spec	1023.	262.6	{238., 282.}	74.3304	0.591496	newly 3 weeks cumul	5532.	1759.28	{1714., 1805.}	68.1981	0.497634	newly 5 weeks spec	383.	67.65	{57., 77.}	82.3368	0.756365	newly 5 weeks cumul	6497.	1958.98	{1908., 2005.}	69.8479	0.52076	newly 53 weeks cumul	7036.	2027.17	{1974., 2082.}	71.1886	0.540517
38	newly 3 weeks spec	915.	949.63	{908., 992.}	4.17377	0.0176012	newly 3 weeks cumul	5124.	4653.82	{4600., 4706.}	9.17603	0.0418135	newly 5 weeks spec	323.	399.55	{377., 431.}	23.6997	0.091827	newly 5 weeks cumul	5962.	5670.78	{5617., 5726.}	4.8846	0.0217617	newly 50 weeks cumul	6391.	6406.6	{6337., 6472.}	0.688155	0.00298265
39	newly 3 weeks spec	1339.	856.27	{822., 884.}	36.0515	0.194375	newly 3 weeks cumul	6703.	4138.03	{4082., 4192.}	38.266	0.209498	newly 5 weeks spec	464.	367.09	{339., 393.}	20.8858	0.1024	newly 5 weeks cumul	7906.	5070.9	{5013., 5126.}	35.8601	0.192888	newly 52 weeks cumul	8666.	5767.35	{5698., 5837.}	33.4485	0.17686
40	newly 3 weeks spec	1822.	939.14	{905., 973.}	48.4555	0.288002	newly 3 weeks cumul	8114.	4420.01	{4367., 4473.}	45.5261	0.263831	newly 5 weeks spec	874.	418.55	{394., 454.}	52.111	0.320307	newly 5 weeks cumul	10 273.	5462.29	{5410., 5516.}	46.8287	0.274336	newly 53 weeks cumul	12 291.	6287.33	{6227., 6359.}	48.8461	0.291136
41	newly 3 weeks spec	362.	83.25	{68., 96.}	77.0028	0.642236	newly 3 weeks cumul	2774.	688.95	{654., 726.}	75.164	0.605289	newly 5 weeks spec	74.	17.41	{11., 23.}	76.473	0.646933	newly 5 weeks cumul	2996.	744.2	{699., 784.}	75.1602	0.605232	newly 56 weeks cumul	3059.	758.51	{720., 797.}	75.204	0.606003
42	newly 3 weeks spec	2612.	1942.34	{1884., 1995.}	25.6378	0.128738	newly 3 weeks cumul	10 679.	7949.14	{7865., 8020.}	25.5629	0.128225	newly 5 weeks spec	1374.	1084.01	{1046., 1122.}	21.1055	0.103138	newly 5 weeks cumul	13 825.	10 487.1	{10 424., 10 562.}	24.1442	0.120018	newly 47 weeks cumul	17 141.	13 612.1	{13 524., 13 691.}	20.5875	0.100116
43	newly 3 weeks spec	2253.	1172.08	{1125., 1226.}	47.9769	0.283999	newly 3 weeks cumul	10 165.	5453.11	{5384., 5520.}	46.3541	0.270483	newly 5 weeks spec	1018.	532.21	{499., 567.}	47.72	0.282151	newly 5 weeks cumul	12 779.	6773.6	{6695., 6855.}	46.9943	0.275692	newly 52 weeks cumul	15 131.	7856.74	{7762., 7958.}	48.0752	0.284644
44	newly 3 weeks spec	1384.	483.13	{457., 508.}	65.0918	0.457417	newly 3 weeks cumul	6755.	2666.44	{2621., 2722.}	60.5264	0.403742	newly 5 weeks spec	626.	165.26	{147., 180.}	73.6006	0.579881	newly 5 weeks cumul	8295.	3112.86	{3065., 3158.}	62.4731	0.425689	newly 52 weeks cumul	9359.	3349.15	{3295., 3409.}	64.2147	0.446333
45	newly 3 weeks spec	1196.	519.48	{493., 543.}	56.5652	0.362434	newly 3 weeks cumul	6409.	3026.09	{2986., 3069.}	52.7837	0.325933	newly 5 weeks spec	536.	170.91	{151., 187.}	68.1138	0.498283	newly 5 weeks cumul	7757.	3494.41	{3448., 3547.}	54.9515	0.346344	newly 54 weeks cumul	8640.	3716.29	{3657., 3767.}	56.9874	0.366432
46	newly 3 weeks spec	1149.	434.58	{409., 461.}	62.1775	0.422797	newly 3 weeks cumul	6165.	2693.43	{2651., 2735.}	56.3109	0.359666	newly 5 weeks spec	377.	128.66	{116., 143.}	65.8727	0.468112	newly 5 weeks cumul	7203.	3057.91	{3010., 3102.}	57.5467	0.372125	newly 56 weeks cumul	7768.	3209.58	{3151., 3265.}	58.682	0.383897
47	newly 3 weeks spec	1358.	512.81	{484., 533.}	62.2378	0.423381	newly 3 weeks cumul	7212.	2557.46	{2500., 2615.}	64.5388	0.4503	newly 5 weeks spec	550.	210.08	{189., 229.}	61.8036	0.41941	newly 5 weeks cumul	8640.	3096.02	{3029., 3168.}	64.1664	0.445766	newly 51 weeks cumul	9536.	3464.44	{3388., 3538.}	63.6699	0.439789
48	newly 3 weeks spec	874.	466.13	{437., 495.}	46.667	0.273514	newly 3 weeks cumul	5250.	2683.32	{2635., 2731.}	48.8891	0.291531	newly 5 weeks spec	248.	152.75	{136., 168.}	38.4073	0.211694	newly 5 weeks cumul	5990.	3103.64	{3051., 3154.}	48.1863	0.285591	newly 54 weeks cumul	6323.	3310.7	{3254., 3364.}	47.6404	0.281039
49	newly 3 weeks spec	879.	503.51	{475., 528.}	42.7179	0.242459	newly 3 weeks cumul	4780.	2379.15	{2331., 2420.}	50.227	0.303047	newly 5 weeks spec	321.	220.49	{200., 239.}	31.3115	0.164047	newly 5 weeks cumul	5641.	2934.67	{2889., 2980.}	47.9761	0.283828	newly 52 weeks cumul	6148.	3368.56	{3313., 3428.}	45.2088	0.261327
50	newly 3 weeks spec	1513.	1028.92	{1000., 1062.}	31.9947	0.167639	newly 3 weeks cumul	7746.	4959.92	{4908., 5005.}	35.968	0.193617	newly 5 weeks spec	636.	441.68	{415., 469.}	30.5535	0.158863	newly 5 weeks cumul	9438.	6075.04	{6022., 6125.}	35.6321	0.191341	newly 53 weeks cumul	10 556.	6905.86	{6830., 6967.}	34.5788	0.184293
